# Association between shift work and insomnia in nurses: a cross-sectional study using the Athens Insomnia Scale

**DOI:** 10.3389/fpubh.2026.1848179

**Published:** 2026-06-03

**Authors:** Mateusz Szczupak, Jacek Kobak, Sabina Krupa-Nurcek

**Affiliations:** 1 Department of Anaesthesiology and Intensive Therapy in the Nicolaus Copernicus Hospital in Gdańsk, Gdańsk, Poland; 2 Department of Otolaryngology, Faculty of Medicine, Medical University of Gdańsk, Gdańsk, Poland; 3 Department of Otolaryngology, University Clinical Center, Gdańsk, Poland; 4 Department of Surgery, Faculty of Medicine, Collegium Medicum, University of Rzeszów, Rzeszów, Poland

**Keywords:** Athens insomnia scale (AIS), insomnia, night work, nurses, sleep

## Abstract

**Introduction:**

Shift work is a recognized occupational risk factor for sleep disturbances, particularly among healthcare professionals. Nurses are especially vulnerable due to irregular work schedules, night duties, and the high physical and psychological demands of clinical practice. The aim of this study was to assess the association between shift work and insomnia among nurses using the Athens Insomnia Scale.

**Methods:**

A cross-sectional survey was conducted among 110 nurses. Data were collected using an anonymous electronic questionnaire consisting of an original sociodemographic and occupational section and the Athens Insomnia Scale (AIS). Statistical analysis was performed using non-parametric methods, including the Mann–Whitney *U*-test, Kruskal–Wallis test, Spearman's rank correlation, and chi-square test, with the significance threshold set at *p* ≤ 0.05.

**Results:**

The mean AIS score in the study group was 9.62 (SD = 4.60), indicating a high burden of sleep disturbances in the analyzed population. Nurses on shift schedules, including night duties, had higher AIS scores than those on daytime schedules only; however, this difference did not reach statistical significance (*p* = 0.204). Similarly, no significant associations were found between AIS score and age, length of service, education level, family status, number of jobs, weekly working hours, or hospital referral level. In contrast, the use of hypnotics was associated with significantly higher insomnia severity (*p* = 0.033).

**Conclusion:**

Insomnia symptoms were common among the nurses in the study group. Although shift work was associated with higher insomnia scores, no statistically significant relationship was observed in this sample. The findings nevertheless indicate the need for further large-scale studies and organizational measures to improve sleep hygiene and working conditions among nurses.

## 1 Introduction

Sleep is defined as a reversible behavioral state with a high arousal threshold (1). Both the quantity and quality of sleep are fundamental to proper physical and mental health. Sleep, a physiological process occurring across many brain centers, is an important element in the proper functioning of all human bodily systems (2, 22). Sleep has been discussed for centuries; however, contemporary medicine defines sleep primarily in terms of behavioral and physiological criteria. It is currently understood as a dynamic neurobiological process essential for physical recovery, cognitive performance, emotional regulation, and metabolic homeostasis. Sleep is therefore not a static behavioral state (1). Nowadays, researchers define sleep based on two criteria: behavioral and physiological. Based on physiological criteria, adult sleep is divided into two independent phases: rapid eye movement (REM) sleep and non-rapid eye movement (NREM) sleep (2, 3). In addition, NREM sleep is divided into three stages: N1, N2, and N3, which are collectively known as slow-wave sleep (SWS). Each stage of sleep is unique and can be visualized in polysomnography (PSG), electroencephalography (EEG), electromyography (EMG) or electrooculogram (EOG), among others (1). Properly modeled sleep includes 4–5 cycles of individual phases, with each phase lasting about 90 min and alternating between light and deep sleep, interrupted by REM sleep.

Sleep disorders are a significant public health problem for the world's population. The loss of the proper process of falling asleep and sleeping due to professional requirements, including shift work, contributes to impaired concentration, attention, and cognitive functions, and may lead to the development of disorders in other physiological processes (4). Studies conducted on sleep in the last decade indicate a link between insufficient or poor quality of sleep and the occurrence of civilization diseases such as obesity, diabetes, cardiovascular diseases, depression, Alzheimer's disease, memory loss or deterioration of attention and focus functions. Lack of sleep is also associated with an increased risk of addiction to alcohol, drugs, increased risky behaviors or aggressiveness, among others (5).

Shift work disrupts the circadian rhythm and impairs physiological recovery processes, which may lead to difficulties with sleep initiation and maintenance, as well as overall deterioration in sleep quality. In the nursing profession, these adverse effects may be intensified by occupational stress, emotional burden, staffing shortages, and prolonged working hours. Therefore, sleep disturbances among nurses should be considered not only an individual health issue but also an important public health concern, as they may affect work performance, decision-making, and patient safety. Although numerous studies have evaluated sleep disturbances among healthcare workers, data focusing on nurses employed in otorhinolaryngology departments remain limited. Moreover, evidence from Central and Eastern European healthcare settings is still insufficient. Therefore, further research is needed to better understand occupational and organizational factors associated with insomnia symptoms in this professional group.

The aim of this study was to assess the prevalence and severity of insomnia symptoms among nurses and to examine their association with selected sociodemographic and occupational factors, with particular emphasis on shift work involving night duties. The study also explored whether age, length of service, education level, family status, weekly working hours, hospital referral level, number of workplaces, and hypnotic use were associated with insomnia severity, as measured by the Athens Insomnia Scale.

## 2 Methods

### 2.1 Sample and recruitment

This cross-sectional study was conducted between August 1, 2023, and September 30, 2023, among nurses employed in otorhinolaryngology departments. Participants were recruited using non-probability convenience sampling. The questionnaire was developed using the Webankieta online survey platform. The survey link was then distributed by e-mail to nurses employed in otorhinolaryngology departments. The platform settings prevented duplicate submissions, thereby minimizing the risk of repeated entries and improving data integrity. Participation was voluntary and anonymous, and respondents were informed of the study's aim before completing the survey. A total of 110 fully completed questionnaires were included in the final analysis.

### 2.2 Study instrument

The research instrument consisted of two components. The first was an original, author-designed questionnaire developed for this study, containing items on sociodemographic and occupational characteristics, including age, sex, family status, education level, length of service, type of workplace, work schedule, weekly working hours, and number of workplaces.

The second component was the Athens Insomnia Scale (AIS), an eight-item self-report instrument developed by Soldatos et al. to assess insomnia symptoms according to ICD-10 criteria. Each item is scored from 0 to 3, yielding a total score of 0–24, with higher scores indicating greater insomnia severity. The Polish version of the AIS has previously demonstrated good psychometric properties and is considered an appropriate screening tool for insomnia symptoms in Polish-speaking populations (19).

### 2.3 Data collection

Before completing the questionnaire, all participants were informed about the study's aim and scope. The questionnaire included information that participation was voluntary and anonymous and that the collected data would be used exclusively for scientific purposes. Respondents were also informed that they could discontinue participation at any stage without providing a reason.

### 2.4 Data analysis

Statistical analysis was performed using IBM SPSS Statistics version 23 and Microsoft Excel 2016. Qualitative variables were presented as counts and percentages, while quantitative variables were reported as means and standard deviations. Due to the non-normal distribution of the analyzed data, non-parametric tests were applied. Differences between two groups were assessed using the Mann–Whitney U-test, whereas comparisons involving more than two groups were performed using the Kruskal–Wallis test. Associations between variables were examined using Spearman's rank correlation coefficients. Chi-square tests were used to analyze qualitative variables. A *p*-value of ≤ 0.05 was considered statistically significant.

### 2.5 Definition of insomnia according to the athens insomnia scale

According to established AIS criteria, a total score of 8 points or higher was interpreted as clinically relevant insomnia symptoms.

### 2.6 Ethical considerations

The study was approved by the Bioethics Committee of the University of Rzeszów, Poland (KBE No. 9/05/2020). The study was conducted in accordance with the principles of the Declaration of Helsinki. To ensure anonymity, no personally identifiable data was collected. Participation was voluntary, and respondents were informed of their right to withdraw from the study at any time without any consequences.

## 3 Results

### 3.1 Characteristics of the research group

A total of 110 nurses participated in the study. The largest age group consisted of respondents aged 46 years or older (58.2%). This category represented late-career nursing staff and was retained as a single group due to the limited sample size in older subgroups. It was followed by respondents aged 36–45 years (19.1%), 26–35 years (15.5%), and 22–25 years (7.3%). Women constituted most of the study population (95.0%). Most respondents worked in one workplace (69.0%), while 26.0% were employed in two workplaces and 5.0% in three workplaces. More than half of the participants (52.0%) reported sleep problems, whereas 14.0% declared the use of hypnotics. A detailed overview is provided in Table 1.

**Table 1:** Baseline demographic and occupational characteristics of the study population.

Variable		*N*	%
Age group	22–25 years	8	7.3
	26–35 years	17	15.5
	36–45 years	21	19.1
	46 years and above	64	58.2
Sex	Female	105	95.5
	Male	5	4.5
Number of workplaces	One	76	69.1
	Two	29	26.4
	Three	5	4.5
Reported sleep problems	Yes	57	51.8
	No	53	48.2
Use of hypnotics	Yes	15	13.6
	No	95	86.4

*N*, number; %, percentage.

Baseline demographic and occupational characteristics are summarized in Table 1. Women constituted most respondents (95.0%), most participants worked at a single workplace (69.0%), and more than half reported sleep problems (52.0%). Hypnotic use was declared by 14.0% of respondents.

### 3.2 Results of the athens insomnia scale

The Athens Insomnia Scale consists of eight items assessing difficulties with sleep induction, nighttime awakenings, early-morning awakening, total sleep duration, overall sleep quality, wellbeing the following day, functional capacity the following day, and daytime sleepiness. Each item is rated on a scale of 0 to 3, yielding a total score of 0–24, with higher scores indicating greater insomnia severity. A score of 8 or more is considered indicative of clinically relevant insomnia symptoms.

In the present study, the mean AIS score was 9.62 (SD = 4.60), indicating a substantial burden of sleep disturbances among the nurses. Reliability analysis demonstrated high internal consistency of the scale, with a Cronbach's alpha coefficient of 0.87, indicating good measurement reliability. Details are presented in Tables 2, 3.

**Table 2:** Descriptive statistics of total Athens Insomnia Scale scores.

Variable	*N*	Min	Max	*M*	SD
Insomnia scale	110	0.00	24.00	9.62	4.60

N – number; min – minimum; max-maximum; M-mean; SD- standard deviation.

**Table 3:** Internal consistency of the Athens Insomnia Scale.

Scale	α	*M*	SD	*N*
Measurement	0.87	1.20	0.85	8

α, Alfa Cronbach's; *M*, mean; SD, standard deviation; *N*, number.

**Figure 1:**
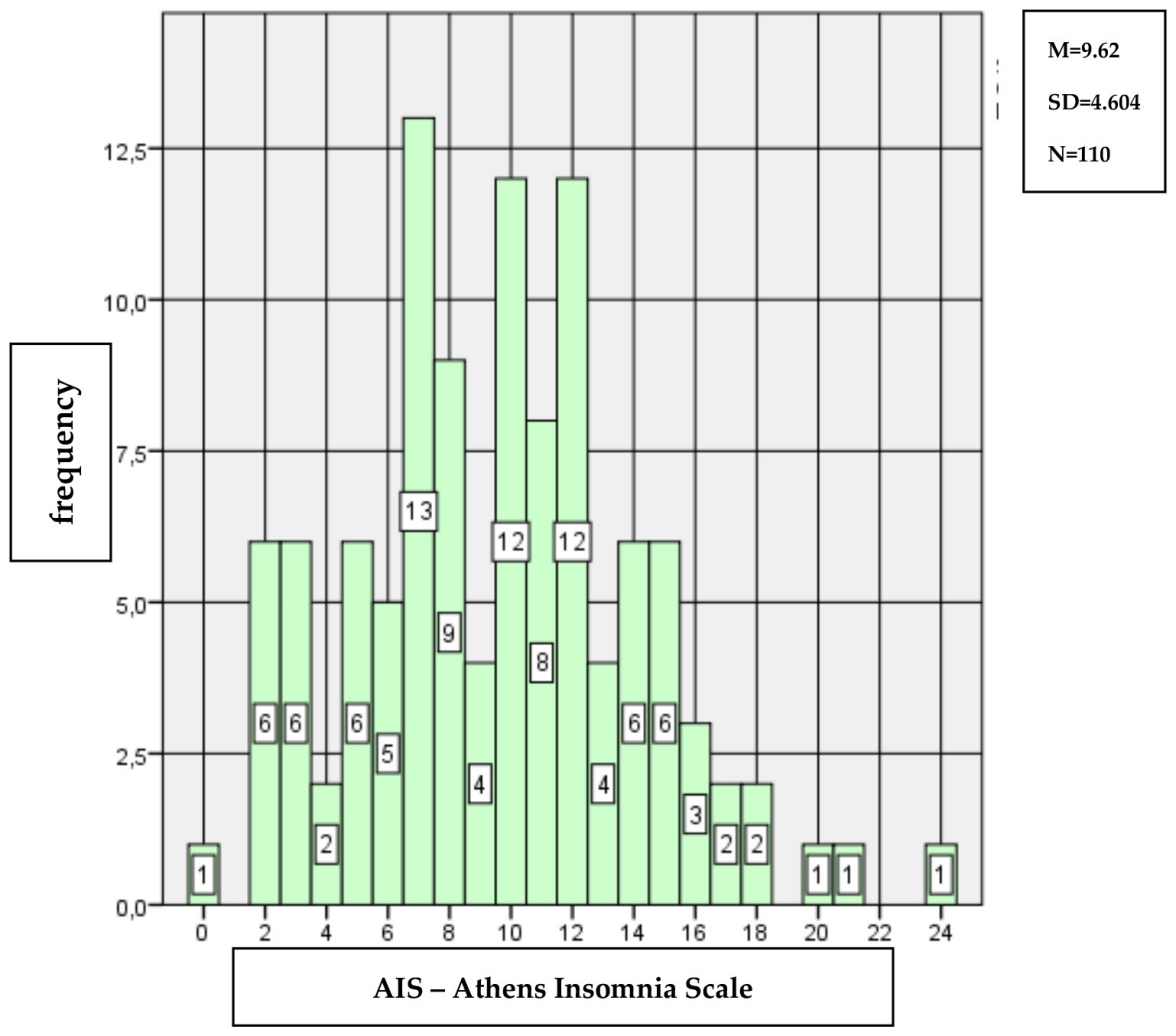
Histogram of the Athens Insomnia Scale; M, means; SD, standard deviation; N, number.

A α factor above 0.70 indicates an acceptable level of measurement accuracy of the analyzed scale; *N* = Number of test items included in the scale construction. The results of the AIS scale are illustrated in Figure 1.

### 3.3 Association between shift work and insomnia

Nurses working shifts including night duties had higher AIS scores than those working only daytime schedules (9.93 ± 4.59 vs. 8.11 ± 4.48). However, the difference did not reach statistical significance (*U* = 704.00; *p* = 0.204). Although shift work was associated with higher insomnia severity in descriptive terms, no statistically significant relationship was observed in the present study (Table 4).

**Table 4:** Comparison of Athens Insomnia Scale scores according to work schedule.

Type of work	Shift work with nighttime			Single shift operation 7 h 35 min			U Mann Whitney test	
	*N*	*M*	SD	*N*	*M*	SD	*U*	*p*
Insomnia scale	91	9.93	4.59	19	8.11	4.48	704.00	0.204

*M*, mean; SD, standard deviation; *N*, number; p, statistical significance.

### 3.4 Association of age, seniority, education, and family status with insomnia severity

#### 3.4.1 Age

No statistically significant associations were found between AIS scores and age (H(3) = 1.82; *p* = 0.611), length of service (H(3) = 2.15; *p* = 0.541), education level (H(2) = 0.06; *p* = 0.972), or family status (H(3) = 4.01; *p* = 0.261). These findings indicate that insomnia severity was comparable across the sociodemographic subgroups analyzed. The results of the analysis are presented in Table 5.

**Table 5:** Athens Insomnia Scale scores across age groups.

Group	*N*	Min	Max	*M*	SD
22–25 years	8	3	16	9.62	4.24
26–35 years	17	2	16	8.71	3.80
36–45 years	21	2	24	9.19	4.77
More than 46 years	64	0	21	10.00	4.83

*N*, number; Min, minimum; Max, maximum; *M*, mean; SD, standard deviation.

#### 3.4.2 Seniority

To evaluate the association between length of service and insomnia severity, a one-way independent-samples analysis was performed. The analysis included *N* = 110 participants divided into four groups according to length of service: < 1 year (*n* = 2; 1.8%), 1–2 years (*n* = 5; 4.5%), 2–5 years (*n* = 12; 10.9%), and >5 years (*n* = 91; 82.7%).

Given the non-normal distribution of the data, a non-parametric Kruskal–Wallis test was applied. The analysis revealed no statistically significant differences in Insomnia Scale scores across the groups defined by length of service, H(3) = 2.15, *p* = 0.541. The effect size was negligible (η^2^H = −0.01), indicating no meaningful association between professional seniority and insomnia severity.

Overall, insomnia severity was comparable across all length-of-service categories. Detailed results are presented in Table 6.

**Table 6:** Athens Insomnia Scale scores according to length of service.

Group	*N*	Min	Max	*M*	SD
2–5 years	12	3	15	9.25	3.47
Less than 1 year	2	11	16	13.50	3.54
More than 1 year	5	6	14	8.60	3.13
More than 5 years	91	0	24	9.64	4.82

Group, independent variable; *N*, number; Min, minimum; Max, maximum; *M*, mean; SD, standard deviation.

#### 3.4.3 Education

To examine the relationship between education level and insomnia severity, a one-way independent-samples analysis was conducted. The analysis included *N* = 105 participants categorized into three groups: medical high school (*n* = 8; 7.6%), first-cycle (bachelor's) degree (*n* = 46; 43.8%), and second-cycle (master's) degree (*n* = 51; 48.6%).

Given the non-normal distribution of the data, a non-parametric Kruskal–Wallis test was applied. The results indicated no statistically significant differences in Insomnia Scale scores across education levels, H(2) = 0.06, *p* = 0.972. The effect size was negligible (η^2^H = −0.02), suggesting no meaningful association between education level and insomnia severity. Overall, insomnia severity was comparable across all education categories.

To assess the association between family status and insomnia severity, a one-way independent-samples analysis was also performed. The analysis included *N* = 110 participants divided into four groups: married (*n* = 69; 62.7%), single (*n* = 12; 10.9%), divorced (*n* = 10; 9.1%), and in a relationship (*n* = 19; 17.3%).

Due to the non-parametric nature of the data, a Kruskal–Wallis test was conducted. The analysis revealed no statistically significant differences in Insomnia Scale scores across family status groups, H(3) = 4.01, *p* = 0.261. The effect size was small (η^2^H = 0.01), indicating a lack of meaningful association between family status and insomnia severity. Insomnia severity was similar across all analyzed family status categories.

### 3.5 Weekly working hours and insomnia

Nurses working more than 40 h per week had slightly higher AIS scores than those working fewer than 40 h per week (9.74 ± 4.84 vs. 9.38 ± 4.14). However, this difference was not statistically significant (*U* = 1346.00; *p* = 0.980). Detailed results are presented in Table 7

**Table 7:** Comparison of Athens Insomnia Scale scores according to weekly working hours.

Variable	More than 40			Less than 40			U Mann–Whitney test	
	*N*	*M*	SD	*N*	*M*	SD	*U*	*p*
Insomnia scale	73	9.74	4.84	37	9.38	4.14	1346.00	0.980

Group, independent variable; N, number; Min, minimum; Max, maximum; M, mean; SD, standard deviation; U, U Mann-Whitney test; p, statistical significance.

### 3.6 Hospital reference level and insomnia

No statistically significant relationship was observed between hospital reference level and the severity of insomnia (H(2) = 2.63; *p* = 0.269). AIS scores were similar in nurses employed in hospitals at reference levels I, II, and III.

### 3.7 Number of jobs, hypnotic use, and insomnia

#### 3.7.1 Number of jobs

No significant correlation was found between the number of workplaces and AIS score (*r* = 0.07; *p* = 0.484). In contrast, nurses who reported using hypnotics had significantly higher AIS scores than those who did not use such medications (12.13 ± 4.19 vs. 9.22 ± 4.56; *U* = 468.50; *p* = 0.033). This finding suggests that hypnotic use was associated with greater insomnia severity in the studied group. The results of the analysis are presented in Tables 8, 9.

**Table 8:** Correlation between number of workplaces and Athens Insomnia Scale score.

Number of jobs	*N*	*r*	*p*
Insomnia scale	108	0.07	0.484

*N*, number; *r*, correlation coefficient; *p*, Statistical significance.

**Table 9:** Comparison of Athens Insomnia Scale scores according to hypnotic use.

Variable	No			Yes			U Mann-Whitney test	
	*N*	*M*	SD	*N*	*M*	SD	*U*	*p*
Insomnia scale	95	9.22	4.56	15	12.13	4.19	468.50	0.033

*U*, U Mann–Whitney test; *p*, statistical significance; *N*, number; *M*, mean; SD, standard deviation.

## 4 Discussion

The present cross-sectional study evaluated insomnia symptoms among nurses employed in otorhinolaryngology departments using the Athens Insomnia Scale. The main findings were as follows: first, insomnia symptoms were common in the analyzed group, as reflected by a mean AIS score above the commonly accepted threshold for clinically relevant insomnia symptoms; second, nurses working shifts that included night duties had higher AIS scores than nurses working only daytime schedules, although this difference did not reach statistical significance; third, the use of hypnotics was associated with significantly higher insomnia severity. No statistically significant associations were observed between AIS score and age, length of service, education level, family status, number of workplaces, weekly working hours, or hospital referral level.

The high burden of insomnia symptoms observed in this study is consistent with the growing body of evidence indicating that sleep disturbances are frequent among nurses and other healthcare professionals. Nurses are a professional group particularly vulnerable to sleep impairment due to irregular working hours, night shifts, heavy emotional workload, responsibility for patient safety, and frequent exposure to stressful clinical situations (21). In a prospective cohort study, Kim and Lee showed that changes in sleep characteristics during the transition to nursing practice may adversely affect nurses' quality of life, suggesting that sleep problems may emerge early in professional life and persist as an occupational health issue (6). Similarly, Membrive-Jiménez et al., in a systematic review and meta-analysis, demonstrated a significant positive relationship between burnout and sleep problems among nurses, emphasizing that poor sleep should be considered not only an individual health problem but also an organizational and occupational safety issue (7, 18).

In our study, the mean AIS score was 9.62, which exceeds the commonly used threshold of 8 points for clinically relevant insomnia symptoms. This finding suggests that a substantial proportion of nurses experienced clinically meaningful sleep disturbances. Similar observations have been reported in studies using the AIS among nursing and healthcare populations. Manzar et al. evaluated the Athens Insomnia Scale among nurses and confirmed its applicability in this occupational group, highlighting that insomnia symptoms can be reliably captured in nursing populations using this instrument (8). In a Polish study by Knap et al., nursing staff working in shift and night systems were found to be particularly exposed to sleep disturbances and related health consequences, supporting the need to evaluate insomnia among nurses working irregular schedules (9). Młynarska et al. also reported that sleep disorders among shift-working nurses were associated with occupational burnout, further indicating that sleep impairment in this group is closely linked to broader psychosocial and occupational strain (10).

Although nurses working shifts that included night duties had higher AIS scores than nurses working only daytime schedules, the difference did not reach statistical significance in the present sample. This result should be interpreted cautiously. The direction of the association is consistent with previous evidence, but the relatively small number of daytime-only workers may have limited the statistical power to detect a significant difference. Chang and Peng, in a systematic review and meta-analysis, found that both rotating shifts and fixed night shifts were associated with poorer sleep quality in nurses compared with fixed daytime work (11). These findings are biologically plausible because night work disrupts circadian synchronization, suppresses nocturnal melatonin secretion, shortens recovery time, and promotes sleep fragmentation. Therefore, the lack of statistical significance in the present study should not be interpreted as evidence that night-shift work is harmless, but rather as a finding that warrants confirmation in larger, more balanced samples.

The absence of significant associations between insomnia severity and age, length of service, education level, family status, number of workplaces, and weekly working hours may suggest that insomnia symptoms among nurses are multifactorial and may not be fully explained by demographic or simple occupational variables alone. Previous studies indicate that psychosocial determinants, including burnout, occupational stress, perceived organizational support, anxiety, and workplace environment, may play an important role in sleep disturbances among nurses (7, 10, 12, 20). Tzenetidis et al. demonstrated an association between effort–reward imbalance and insomnia among healthcare personnel, suggesting that perceived imbalance between occupational demands and rewards may contribute to sleep impairment (12). This may partly explain why variables such as age or education level were not significantly associated with AIS scores in our cohort, whereas broader psychosocial factors not captured in the present study may have influenced insomnia severity.

The association between hypnotic use and higher AIS scores is clinically important. In the present study, nurses who reported using hypnotics had significantly higher insomnia severity than those who did not use such medications. This finding most likely reflects reverse causality: individuals with more severe insomnia symptoms are more likely to seek pharmacological sleep support. Forthun et al., in a large cross-sectional study of Norwegian nurses, reported that 7.5% used prescribed sleep medication, while additional respondents used over-the-counter sleep medication or melatonin (13). The higher proportion of hypnotic use in our sample may reflect differences in study population, working conditions, cultural patterns of medication use, or the higher burden of insomnia symptoms observed in the analyzed group. Importantly, the association between hypnotic use and AIS score should not be interpreted as evidence that hypnotics increase insomnia severity; rather, it indicates that hypnotic use may serve as a marker of more severe or persistent sleep problems.

The present findings also align with evidence from studies conducted among healthcare workers during and after the COVID-19 pandemic, which consistently showed a high prevalence of insomnia symptoms in medical personnel. Sahebi et al., in an umbrella review of meta-analyses, reported a high prevalence of insomnia among healthcare workers during the pandemic, highlighting this professional group's vulnerability to sleep disturbances under increased workload and psychological stress (14). In Polish nurses, Rachubińska et al. found that insomnia symptoms were part of a broader pattern of psychological distress involving anxiety, depression, stress, and traumatic event exposure (15). Although the present study was not designed to evaluate pandemic-related stressors, these findings are relevant because they show that insomnia in nurses often coexists with psychological and occupational strain.

The relationship between insomnia and mental health should also be considered. Sikaras et al. reported high levels of insomnia among nurses 2 years after the onset of the pandemic crisis and showed significant associations between insomnia, anxiety, and perceived family support (16). These findings support the view that insomnia among nurses is embedded in a broader psychosocial context and may be influenced by both workplace and non-workplace factors. This is particularly important for interpreting the present results, because the study did not include measures of anxiety, depression, burnout, perceived stress, resilience, or family support. Therefore, the lack of significant associations between AIS score and several demographic or occupational variables should be interpreted in the context of unmeasured psychosocial determinants.

From an occupational health perspective, sleep disturbances among nurses are clinically and organizationally relevant. Poor sleep may contribute to daytime fatigue, impaired concentration, reduced vigilance, emotional dysregulation, decreased work performance, and increased risk of errors. Gómez-García et al. showed that nurses' sleep quality was associated with work environment and perceived quality of care, suggesting that sleep problems may have consequences beyond individual wellbeing (17). Therefore, addressing insomnia symptoms among nurses should be regarded as part of a broader patient safety and workforce sustainability strategy. Potential interventions include optimizing shift schedules, limiting consecutive night-duty periods, ensuring adequate recovery time between shifts, improving staffing levels, and providing access to non-pharmacological insomnia interventions, such as sleep hygiene education and cognitive-behavioral strategies.

The specificity of the present study lies in its focus on nurses employed in otorhinolaryngology departments. Although sleep disturbances among nurses have been widely investigated in general hospital settings, data regarding specific clinical departments remain limited. Otorhinolaryngology nursing may involve exposure to patients with airway problems, postoperative monitoring needs, bleeding risk, tracheostomy care, and acute clinical situations requiring sustained vigilance. These occupational features may contribute to psychological and physical workload, although this hypothesis requires further study. The present findings, therefore, add preliminary data from a specific clinical nursing subgroup and from a Central and Eastern European healthcare context.

This study has several practical implications. First, routine screening for insomnia symptoms among nurses may help identify individuals requiring early support. The AIS is short, easy to administer, and suitable for occupational screening. Second, hypnotic use among nurses should prompt careful clinical and occupational assessment, as it may indicate more severe insomnia rather than simply isolated medication use. Third, organizational interventions should not focus solely on individual sleep hygiene but should also address shift design, recovery opportunities, workload, staffing, and psychosocial support. Finally, future studies should use larger multicenter samples and include validated measures of burnout, occupational stress, anxiety, depression, resilience, and perceived workplace support to better explain the determinants of insomnia among nurses.

In summary, the present study confirms that insomnia symptoms are common among nurses employed in otorhinolaryngology departments. Although shift work, including night duties, was associated with higher AIS scores in descriptive terms, the association was not statistically significant in this sample. Hypnotic use was significantly associated with greater insomnia severity. These findings are consistent with current literature indicating that sleep disturbances among nurses are frequent, multifactorial, and closely linked to occupational and psychosocial conditions. Larger prospective studies are needed to clarify causal pathways and to develop effective preventive and organizational interventions.

## 5 Conclusion

Insomnia symptoms were common among the nurses in the study, as reflected by a mean AIS score above the diagnostic threshold. Although nurses working shifts, including night duties, had higher insomnia scores than those working only daytime schedules, this relationship was not statistically significant in the present sample. The use of hypnotics was associated with significantly greater insomnia severity. Overall, the findings suggest that sleep disturbances among nurses are an important occupational and public health issue and support the need for further large-scale studies and interventions to improve work organization and sleep hygiene.

### 5.1 Implications for practice

Healthcare institutions should consider implementing organizational strategies to reduce the burden of sleep disturbances among nurses. Such measures may include improved scheduling practices, limiting consecutive night shifts, ensuring sufficient recovery time between shifts, and educational interventions focused on sleep hygiene. Where appropriate, access to psychological support and non-pharmacological management of insomnia should also be strengthened.

### 5.2 Study limitations

This study has several limitations. First, the sample size was relatively small, which may have reduced the statistical power to detect significant associations. Second, the study was based on self-reported data, which may be subject to recall bias and subjective interpretation. Third, the cross-sectional design does not allow for causal conclusions. Finally, the study did not include potentially relevant psychological and environmental variables, such as perceived stress, burnout, workload, workplace support, or access to mental health resources, which may also influence sleep quality. Future studies should include larger and more diverse samples and incorporate a broader range of occupational and psychosocial determinants.

In addition, although multivariable regression modeling would provide further insight into independent predictors of insomnia, the archived dataset available for revision contained aggregated outputs rather than participant-level raw data, which precluded reliable *post hoc* model reconstruction.

## Data Availability

The original contributions presented in the study are included in the article/supplementary material, further inquiries can be directed to the corresponding author.

## References

[1] Sushil K. Jha, Vibha M Jha. Sleep, Memory And Synaptic Plasticity. Singapore: Springer (2019) 2–3.

[2] Baranwal N Yu K Ph Siegel SN. Sleep physiology, pathophysiology and sleep hygiene. Prog Cardiovasc Dis. (2023) 77. 59–69. doi: 10.1016/j.pcad.2023.02.005 36841492

[3] Oriano M. Clinical Electroencephalography. Cham: Springer (2016) 153–4.

[4] Chokroverty S Strambi-Ferini L. Oxford Textbook of Sleep Disorders. New York, NY: Oxford University Press (2017) 13–4. doi: 10.1093/med/9780199682003.001.0001

[5] Kothare SV Scott-Quattrucci R. Sleep Disorders In Adolescentes. A clinicam casebook. Cham: Springer (2017) 1–2.

[6] Kim K Lee Y. Influence of sleep characteristic changes on nurses' quality of life during their transition to practice: a prospective cohort study. Int J Environ Res Public Health. (2022) 19:573. doi: 10.3390/ijerph19010573 35010831 PMC8744848

[7] Membrive-Jiménez MJ Gómez-Urquiza JL Suleiman-Martos N Velando-Soriano A Ariza T De la Fuente-Solana EI . Relation between burnout and sleep problems in nurses: a systematic review with meta-analysis. Healthcare. (2022) 10:954. doi: 10.3390/healthcare10050954 35628091 PMC9140410

[8] Manzar MD Albougami A Hassen HY Sikkandar MY Pandi-Perumal SR Bahammam AS. Psychometric validation of the athens insomnia scale among nurses: a robust approach using both classical theory and rating scale model parameters. Nat Sci Sleep. (2022) 14:725–39. doi: 10.2147/NSS.S325220 35478720 PMC9035448

[9] Knap M Maciag D Trzeciak-Bereza E Knap B Czop M Krupa S. Sleep disturbances and health consequences induced by the specificity of nurses' work. Int J Environ Res Public Health. (2022) 19:9802. doi: 10.3390/ijerph19169802 36011431 PMC9408457

[10] Młynarska A Bronder M Kolarczyk E Manulik S Młynarski R. Determinants of sleep disorders and occupational burnout among nurses: a cross-sectional study. Int J Environ Res Public Health. (2022) 19:6218. doi: 10.3390/ijerph19106218 35627754 PMC9140934

[11] Chang WP Peng YX. Influence of rotating shifts and fixed night shifts on sleep quality of nurses of different ages: a systematic literature review and meta-analysis. Chronobiol Int. (2021) 38:1384–96. doi: 10.1080/07420528.2021.1931273 34056959

[12] Tzenetidis V Papathanasiou I Tzenetidis N Nikolentzos A Sarafis P Malliarou M. Effort reward imbalance and insomnia among greek healthcare personnel during the outbreak of COVID-19. Mater Sociomed. (2021) 33:124–30. doi: 10.5455/msm.2021.33.124-130 34483741 PMC8385725

[13] Forthun I Waage S Pallesen S Moen BE Bjorvatn B. Sleep medication and melatonin use among Norwegian nurses - a cross-sectional study. Nurs Open. (2022) 9:233–44. doi: 10.1002/nop2.1057 34534412 PMC8685790

[14] Sahebi A Abdi K Moayedi S Torres M Golitaleb M. The prevalence of insomnia among health care workers amid the COVID-19 pandemic: an umbrella review of meta-analyses. J Psychosom Res. (2021) 149:110597. doi: 10.1016/j.jpsychores.2021.110597 34388380 PMC8443320

[15] Rachubińska K Cybulska AM Sołek-Pastuszka J Panczyk M Stanisławska M Ustianowski P . Assessment of psychosocial functioning of polish nurses during COVID-19 pandemic. Int J Environ Res Public Health. (2022) 19:1435. doi: 10.3390/ijerph19031435 35162456 PMC8835236

[16] Sikaras C Tsironi M Zyga S Panagiotou A. Anxiety, insomnia and family support in nurses, two years after the onset of the pandemic crisis. AIMS Public Health. (2023) 10:252–67. doi: 10.3934/publichealth.2023019 37304592 PMC10251058

[17] Gómez-García T Ruzafa-Martínez M Fuentelsaz-Gallego C Madrid JA Rol MA Martínez-Madrid MJ . Nurses' sleep quality, work environment and quality of care in the Spanish National health system: observational study among different shifts. BMJ Open. (2016) 6:e012073. doi: 10.1136/bmjopen-2016-012073 PMC4985858 27496241

[18] Parada ME Moreno R Mejías MZ Rivas FA Rivas FF Cerrada SJ . Job satisfaction and burnout syndrome in the nursing staff of the Instituto Autónomo Hospital Universitario Los Andes, Mérida, Venezuela. Rev Fac Nac. (2005) 23:33–45.

[19] Fornal-Pawłowska M Wołyńczyk-Gmaj D Szelenberger W. Walidacja Ateńskiej Skali Bezsenności. [Validation of the polish version of the athens insomnia scale]. Psychiatr Pol. (2011) 45:211−21. 21714210

[20] Gómez-Urquiza JL Monsalve-Reyes CS San Luis-Costas C Fernández-Castillo R Aguayo-Estremera R. Cañadas-de la Fuente GA. Factores de riesgo y niveles de burnout en enfermeras de atención primaria: Una revisión sistemática. Aten Primaria. (2017) 49:77–85. 27363394 10.1016/j.aprim.2016.05.004 PMC6876264

[21] Gallagher RM Gormley DK. Perceptions of stress, burnout, and support systems in pediatric bone marrow transplantation nursing. Clin J Oncol Nurs. (2009) 13:681–5. doi: 10.1188/09.CJON.681-685 19948465

[22] Darroff RB Fenichel GM Jankovic J Mazziotta JC. Neurology In Clinical Practice. 6th Edn. (2012) 2:1634–9. doi: 10.1016/B978-1-4377-0434-1.00001-3

